# Basal ganglia calcification and novel compound heterozygous mutations in the PANK2 gene in a Chinese boy with classic Pantothenate kinase-associated neurodegeneration: A case report

**DOI:** 10.1097/MD.0000000000010316

**Published:** 2018-04-13

**Authors:** Xulai Shi, Feixia Zheng, Xiuyun Ye, Xiucui Li, Qianlei Zhao, Zhongdong Lin, Ying Hu, Jiwen Wang

**Affiliations:** a Department of Neurology, Children's Medical Center, Qilu Hospital of Shandong University, Brain Science Research Institute, Shandong University, Jinan, Shandong; b Department of Pediatric Neurology, the Second Affiliated Hospital and Yuying Children's Hospital, Wenzhou Medical University, Zhejiang; c Department of Neurology, Shanghai Children's Medical Center, Shanghai Jiaotong University School of Medicine, Shanghai, China.

**Keywords:** calcification, heterozygous mutation, PANK2 gene mutation, pantothenate kinase-associated neurodegeneration

## Abstract

**Rationale:**

Pantothenate kinase-associated neurodegeneration (PKAN) represents an autosomal recessive hereditary disease. In this report, a PANK2 gene mutation in a Chinese child was identified, as well as detections of PKAN among his family members. Our findings exposed a world-wide novel compound heterozygous mutation.

**Patient concerns:**

We described a 6-year-old male patient with PKAN, exhibiting involuntary movement for a period of 1.5 years, as well as feeding difficulties for 2 weeks.

**Diagnosis:**

Due to brain computed tomography and magnetic resonance imaging results, and patient behavior, the attending physician suspected a hereditary effect.

**Interventions:**

The patient sample underwent high-throughput sequencing. Subsequently, his parents and sister were screened for the mutations identified in the patient genome.

**Outcomes:**

High-throughput sequencing revealed a novel complex heterozygous mutation of the PANK2 gene, which was detected in the second and fourth exons, c.A650G, and c.T1341G, respectively, resulting in amino acid alterations (p.D217G and p.D447E, respectively). The child's father was confirmed to possess a heterozygous c.A650G mutation, while his mother was heterozygous for the c.T1341G mutation.

**Lessons:**

The key finding of the study encompassed the detection of a novel PANK2 gene mutation in a child of Chinese ethnicity with PKAN. The PANK2 gene c.A650G, as well as c.T1341G, mutations may be potential mutation hotspots in children with PKAN in Mainland China.

## 1 Introduction

Pantothenate kinase-associated neurodegeneration (PKAN) represents an autosomal recessive hereditary disease in which iron accumulates in the basal ganglia, leading to progressive dystonia, spasticity, parkinsonism, neuropsychiatric abnormalities, and optic atrophy or retinal degeneration.^[1]^ However, the condition remains one of the principal neurodegenerative diseases manifested by brain iron accumulation. Neurodegeneration with brain iron accumulation (NBIA), previously known as Hallervorden-Spatz syndrome, has been reported in recent years to be associated with PANK2 disease-causing genes, located at 20 p12. 3–13.^[2,3]^ Until now, there have been approximately 120 mutations (Human Gene Mutation Databases) confirmed in the PANK2 gene, including approximately 80 missense and nonsense mutations.^[4]^ During this study, a PANK2 gene mutation in a Chinese child was identified, as well as detections of PKAN among his family members. The aforementioned findings were indicative of a novel compound heterozygous mutation.

## 2 Case report

**Figure 1:**
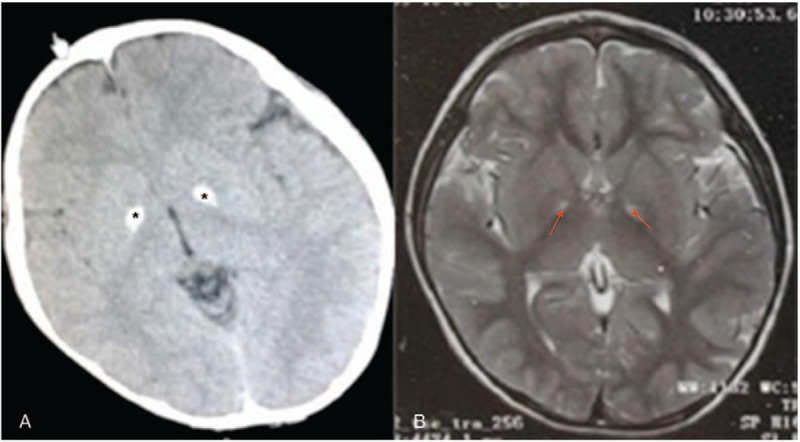
Brain CT scan displayed a bilateral calcification of the globus pallidus (A, black star) and axial T2 weighted brain MRI displaying typical “eye of the tiger” (B, red arrow). CT = computed tomography; MRI = magnetic resonance imaging.

A 6-year-old male, exhibiting involuntary movement for a period of 1.5 years, as well as feeding difficulties for a period of 2 weeks, was hospitalized for the third time on the 24th of December 2016. The child was delivered at full-term, and no asphyxia was observed at birth. Before the age of 10 months, the child displayed normal development. The parents’ marriage was non-consanguineous, generally healthy, and did not show gait difficulty or dysarthria. Three years before presentation, the frequent emergence of fever, approximately once per month with temperatures ranging between 38.0° and 39.0° Celsius, lasted for 3 days before returning to normal after physical cooling or paracetamol treatment. The involuntary movements displayed by the 4.5-year-old child were observed predominantly in the thumb of his right hand, which he repeatedly placed in his mouth. Moreover, slight involuntary limb movements gradually increased, accompanied by choreiform movement, and dysphagia. The aforementioned involuntary movements intensified during fever, fatigue, and tension, but reduced or ceased during sleep. Furthermore, we observed diminished active motion from the child. He was unable to stand or walk, and exhibited progressive backward language formation, which resulted in a distinct reduction in his ability to communicate. Despite this speech impediment, the child was able to comprehend information conveyed to him. Additionally, the child struggled to see in poorly lit areas, easily fell during toe-waling, had poor physical fitness, exhibited mental intellectual disability retardation and dysarthria, as well as frequently communicating in sentences limited to 10 words or less. The child had previously undergone rehabilitation therapy, but the effect was minimal. Upon admission to the hospital, a physical examination assessed normal vital signs, normal skin color, and no abnormalities of the heart, lung, and abdomen. An examination of the nervous system revealed: clear mind, insistinct speech, and the cranial nerves (–), corneal K-F ring (–), and fundus were normal. Neck rigidity (+), pupillary light reflex, eye movements, and finger-nose test results were normal. His limb muscle strength was grade IV, muscle tension increased paroxysmal, he was unable to stand and walk, and exhibited involuntary movement of the limbs, double tendon reflex (+ + +), and an asymmetric Babinski reflex (+), which was present on the left. The auxiliary examination detected the bilateral basal ganglia symmetry had a high density of patchiness in a brain computed tomography (CT) scan without contrast (Fig. 1A), and no obvious abnormalities of the head in an magnetic resonance imaging (MRI) test (Fig. 1B). Cerebrospinal fluid examination revealed that the standard immunoglobulin antibodies, IgG, IgM, and IgA, were normal. Liver and kidney function, electrolytes, blood ammonia, blood lactates were all normal. T-cell subsets were observed to have normal immune function. Complement, ceruloplasmin (–). The tandem mass spectrometry data obtained revealed that 3-hydroxybutyric acid was significantly increased, which is in agreement with observations of inherited metabolic diseases, and thus suggests ketosis. Furthermore, free carnitine increased in blood, while C18 decreased. The antinuclear antibody profile was normal, and anticardiolipin antibody and anti-O levels were insignificant (–). The child was administered various combinations of trihexyphenidyl, clonazepam, tiapride, haloperidol, baclofen, and amantadine hydrochloride, as well as an assortment of vitamin pills and a high-dose intravenous immunoglobulin therapy. Short-term involuntary movement symptoms appeared to be slightly improved, but the overall trend was progressive deterioration, and the child's language ability gradually declined. The child had a healthy younger sister, who was confirmed to have no abnormalities after testing for the PKAN gene.

## 3 Sequencing detection

Gene capture high-throughput sequencing detection was conducted as follows: extraction of DNA from peripheral blood post-fragmentation, library preparation, probe capture gene panel exon, as well as in the adjacent area, and finally, sent to the high-throughput sequencing platform for sequencing. The samples in the DNA sequence were then measured in the target area.

## 4 Detection results

In the obtained sample, 2 heterozygous mutations, both of which are missense mutations, were discovered in the gene, c.A650G (adenine> guanine), which generated a p.D217G (aspartic acid > glycine) amino acid change; c.T1341G (thymine>guanine), resulted in a p.D447E (aspartic acid> glutamic acid) amino acid change. The 2 sites have yet to be reported in the HGMD database (Table 1). We confirmed that the proband's sister did not possess any of the child's mutations. Upon examination, his parents and sister did not exhibit any neurological abnormalities.

**Table 1:**
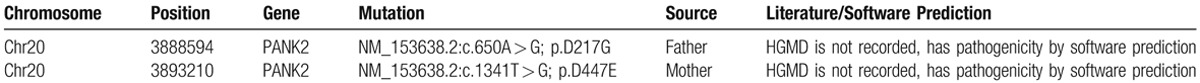
Gene capture high-throughput sequencing detection results.

## 5 Discussion

The incidence of PKAN has been reported to be approximately 3 in 1,000,000.^[5]^ To date, a lack of detailed epidemiological data regarding this disorder remains. Two subtypes are known, classical and non-classical,^[5]^ which correspond to age of onset and progression of PKAN. The clinical manifestations of PKAN are generally extrapyramidal: dystonia, gait irregularity, athetosis, walking instability, limb weakness and spasms, numbness, ataxia, language disorders, slurred speech, dementia, dizziness, headache, disturbance of consciousness, coughing whereas consuming water, and eating difficulties.^[4,5]^ The pathogenesis of PKAN is putatively initiated by inhibition of pantothenate kinase 2 synthesis reportedly due to a PANK2 mutation. Therefore, 4-phosphopantothenate cysteine syntheses are limited, including cysteine and its intermediate product aggregation, and the iron involved in the aggregation process. In our study, we found that PANK2 is associated with neurodegenerative disorders that involve pantothenate kinase. The deposition of iron in the globus pallidus, substantial nigra, and red nucleus, lead to increases in the production of free radicals and membrane protein synthesis disorders, thus ultimately resulting in neuronal degeneration.^[5–8]^ Cranial MRI verified symmetric “eye of the tiger sign” in bilateral basal ganglia. After 10-months-old in age, the child exhibited slowed movement and language development, as well as poor rehabilitation training. At 4.5 years of age, his involuntary movements and other extrapyramidal symptoms were observed to be progressively worse, and he displayed poor response to drug therapy treatments. The child's disorder is categorized under the classic subtype, which is consistent with relevant clinical studies.^[5–8]^ Despite the patient experiencing vision problems, no abnormalities were observed upon examination of the fundus, which may be due to his young age according to developmental specifications. The child was confirmed to have both the typical MRI features (“eye of the tiger”) and basal ganglia calcifications. Moreover, the brain CT scan exhibited more indistinguishable abnormalities than the MRI, highlighting the value of brain CT in early diagnosis of PKAN.^[7,8]^ In our case study, two heterozygous mutations of the PANK2 gene were detected and found to represent missense mutations. The detection of the 2 samples of heterozygous point mutations in the PANK2 gene were found in the parents, the father's mutation, c.A650G (adenine >guanine), resulted in the amino acid change p.D217G (aspartic acid > glycine), while the mother's mutation, c.T1341G (thymine >guanine), produced the amino acid alteration p.D447E (aspartic acid > glutamic acid). The HGMD database is yet to report these 2 sites. Until now, this mutation has not been reported to lead to PKAN, thus the compound heterozygous mutation reported in this study is likely to represent a new pathogenic mutation of PKAN.^[9,10]^

## Acknowledgments

We would like to thank the patient and his family for accepting our invitation to participate in this study.

## Author contributions

**Data curation:** Xulai Shi, Zhongdong Lin.

**Formal analysis:** Xulai Shi, Feixia Zheng, Ying Hu.

**Investigation:** Xulai Shi, Qianlei Zhao, Zhongdong Lin.

**Methodology:** Xiuyun Ye.

**Project administration:** Xiucui Li.

**Supervision:** Jiwen Wang.

**Validation:** Jiwen Wang, Feixia Zheng, Qianlei Zhao.

**Writing – original draft:** Jiwen Wang, Xulai Shi.

**Writing – review & editing:** Jiwen Wang, Xulai Shi.
